# Antioxidant Functions of Vitamin D and CYP11A1-Derived Vitamin D, Tachysterol, and Lumisterol Metabolites: Mechanisms, Clinical Implications, and Future Directions

**DOI:** 10.3390/antiox13080996

**Published:** 2024-08-17

**Authors:** Héctor Vázquez-Lorente, Lourdes Herrera-Quintana, Laura Jiménez-Sánchez, Beatriz Fernández-Perea, Julio Plaza-Diaz

**Affiliations:** 1 Department of Physiology, Schools of Pharmacy and Medicine, University of Granada, 18071 Granada, Spain; hectorvazquez@ugr.es (H.V.-L.); lourdesherrera@ugr.es (L.H.-Q.); laurajimsan@gmail.com (L.J.-S.); 2 Biomedical Research Center, Health Sciences Technology Park, University of Granada, 18016 Granada, Spain; 3 Instituto de Investigación Biosanitaria IBS.GRANADA, Complejo Hospitalario Universitario de Granada, 18014 Granada, Spain; 4 Immunology and Clinical Analysis Service, Virgen de las Nieves University Hospital, 18014 Granada, Spain; beafdezperea@correo.ugr.es; 5 Department of Biochemistry and Molecular Biology II, School of Pharmacy, University of Granada, Campus de Cartuja s/n, 18071 Granada, Spain; 6 Children’s Hospital of Eastern Ontario Research Institute, Ottawa, ON K1H 8L1, Canada

**Keywords:** vitamin D, antioxidant, oxidative stress, reactive oxygen species, neurodegenerative diseases

## Abstract

Evidence is increasing that vitamin D and CYP11A1-derived vitamin D, tachysterol, and lumisterol metabolites play a significant antioxidant role beyond its classical functions in bone health and calcium metabolism. Several recent studies have linked these elements to reduced oxidative stress as well as improved immune, cardiovascular, and neurological functions as a result of chronic kidney disease and cancer. Additionally, supplementation with this vitamin has been shown to be one of the most cost-effective micronutrient interventions worldwide, highlighting its potential as a therapeutic approach. The underlying mechanisms and implications of this antioxidant function of vitamin D or CYP11A1-derived vitamin D, tachysterol, and lumisterol metabolites are not well understood. This comprehensive and narrative review is aimed at summarizing the current evidence regarding the molecular mechanisms implicated in this antioxidant function of vitamin D, as well as to provide a general overview and to identify key research areas for the future, offering an extensive perspective that can guide both researchers and clinicians in the management of diseases associated with oxidative stress and/or insufficient vitamin D status.

## 1. Introduction

Vitamin D, historically considered an essential nutrient derived from various dietary sources, is now defined as a steroid hormone. Its precursor molecule is synthesized in the skin when exposed to ultraviolet radiation (UVR) [1], which further generates electrical, chemical, and biological signals that are transmitted to the brain, endocrine, and immune systems, as well as other central organs. These systems work in concert to regulate body homeostasis, thereby providing a photo-neuro-immuno-endocrinological perspective [2]. Among its classical functions, vitamin D has been recognized as an important factor in the regulation of neuro-musculoskeletal activities, tightly controlling circulating Ca^2+^ levels. However, in recent years, numerous other physiological processes have been found to be influenced by vitamin D, with significant implications in the prevention and reduction of severity of various conditions such as autoimmune, cardiovascular, and metabolic disorders [1,3]. Due to the pleiotropic vitamin D effects and the high prevalence of its insufficient status worldwide, supplementation with this vitamin has been shown to be one of the most cost-effective micronutrient interventions [4]. Furthermore, among its various beneficial effects, vitamin D has been observed to reduce levels of oxidative stress (OS) parameters in different diseases, such as polycystic ovary syndrome [5] and mild cognitive impairment [6].

As a result of reactive oxygen species (ROS), reactive nitrogen species (RNS), and other free radicals, OS contributes to the onset and progression of various diseases, such as diabetes, obesity, neurological disorders, and cardiovascular conditions [7]. The imbalance between pro-oxidant and antioxidant molecules leads to cellular damage, which manifests as inflammation, DNA damage, protein cross-linking, lipid membrane peroxidation, mitochondrial dysfunction, and necrosis/apoptosis [8]. Therefore, maintaining an adequate concentration of prooxidants and antioxidants, which constitute the redox systems, is crucial. Disruption of this equilibrium can contribute to the development of pathological conditions such as cancers and chronic diseases [9]. In this context, the use of pharmacological antioxidants for health protection has emerged as a promising therapeutic target in recent decades. Consequently, dietary recommendations emphasizing the consumption of plant foods rich in antioxidants and the use of antioxidant supplements have garnered increasing interest [10]. Regarding vitamin D supplementation and its effect on antioxidant status, while several studies indicate that vitamin D decreases OS parameters, the results in human studies remain controversial. Thus, the antioxidant function of vitamin D and its underlying mechanisms are not yet well established [11].

Based on the above considerations, the aim of the present study is to review the current evidence regarding the main antioxidant functions of vitamin D and CYP11A1-derived vitamin D, tachysterol, and lumisterol metabolites, and their role in OS. This review provides novel insights into its mechanisms, clinical implications, and future perspectives.

## 2. Oxidative Stress and Antioxidant Status

OS is defined as a dysregulation between the production of RNS and ROS, and the endogenous antioxidant defense mechanisms, collectively known as the redox state [12]. OS indicates a disparity between ROS generation and the organism’s capacity to detoxify reactive intermediates or repair the resulting damage through an adequate antioxidant defense system. High levels of ROS can damage nucleic acids, proteins, membranes, nucleic acids, organelles, and lipids, eventually leading to irreversible cell damage and death [13]. This macromolecular damage is often considered in terms of oxidative mechanisms linked to free radicals, which are reactive and can initiate chain reactions that propagate to damage multiple molecules [14]. Free radicals or active oxidants, such as superoxide radicals (O2^•−^), hydroxyl radicals (^•^OH), and hydrogen peroxide (H_2_O_2_), which are originated from oxygen, are collectively termed ROS [15]. It is widely accepted that the principal source of free radicals in the cell is the mitochondrial respiratory chain [16]. The production of reactive species can result from biochemical processes, exposure to damaging agents (e.g., environmental pollutants and radiation), or limited capabilities of endogenous antioxidant systems [17].

ROS are implicated in various biological functions, including the regulation of physiological processes and the pathogenesis of numerous human diseases [18]. Under normal conditions, there is a balance between oxidant and antioxidant activity due to the action of the antioxidant defense system. RNS and ROS are neutralized by both extrinsic and intrinsic methods. Intrinsic mechanisms mainly involve enzymatic processes [19], including antioxidant enzymes such as superoxide dismutase (SOD), peroxidases, and glutathione reductase (GR). These enzymes are distributed through the cell to protect DNA integrity, hormones, enzymes, cell membranes, and other organelles and structures from ROS-induced damage [20,21].

## 3. Background and Significance of Vitamin D in Human Health

### 3.1. Sources, Synthesis, and Metabolism

Vitamin D is a fat-soluble secosteroid that can be obtained exogenously through diet or supplements. The following foods are good sources of vitamin D: fatty fish (such as salmon, mackerel, and sardines), fish liver oils, fortified foods (such as milk, orange juice, cereals, and egg yolks), and fortified foods (such as milk, orange juice, and cereals [22]. Vitamin D supplements are also an effective means of ensuring adequate intake, particularly for individuals at deficiency risk; in particular, those with limited sun exposure, the elderly, and people with darker skin should avoid excessive exposure to the sun [23]. 

Endogenously, pre-vitamin D_3_ can be synthesized in the skin following the absorption of ultraviolet-B (UVB) energy by 7-dehydrocholesterol (7DHC) [24,25]. Prolonged exposure of 7-DHC to UVB results in the phototransformation of pre-vitamin D_3_ into tachysterol and lumisterol [26]. Specifically, in the skin, the absorption of UVB energy leads to the breakage of the unsaturated B ring in 7DHC, causing its transformation into corresponding secosteroids, with or without a full-length side chain. This results in the formation of vitamin D-like, tachysterol-like, and lumisterol-like compounds when the broken B ring reseals in a different stereochemical configuration [27]. Although the role of vitamin D_3_ as a prohormone is well-established, tachysterol and lumisterol have been reported to exhibit similar functions [28].

Vitamin D activation occurs through two distinct pathways: the canonical and non-canonical mechanisms. Concerning the canonical pathway [29], vitamin D, in its inactive form, undergoes specific metabolic processes to become active and is transported to the liver, bound to vitamin D-binding proteins, where it undergoes hydroxylation to form 25-hydroxyvitamin D. This metabolite is used to assess a person’s vitamin D status [30]. Various tissues, including prostate, pancreatic islets, breast, colon, malignant and immune cells, macrophages, vascular smooth muscle cells, and especially the kidneys, possess 1-α-hydroxylase [31]. In these tissues, 25-hydroxyvitamin D undergoes a second hydroxylation to produce 1,25-dihydroxycholecalciferol, the physiologically active form of vitamin D. This active form exerts its effects by interacting with vitamin D receptors (VDR) [32], which then enter the nucleus and heterodimerize with the retinoic acid X receptor [33]. This receptor complex acts on vitamin D-response elements to exert transcriptional control over a set of genes [34], thereby increasing the transcription of vitamin D-dependent genes, which are essential for its various functions. 

**Figure 1:**
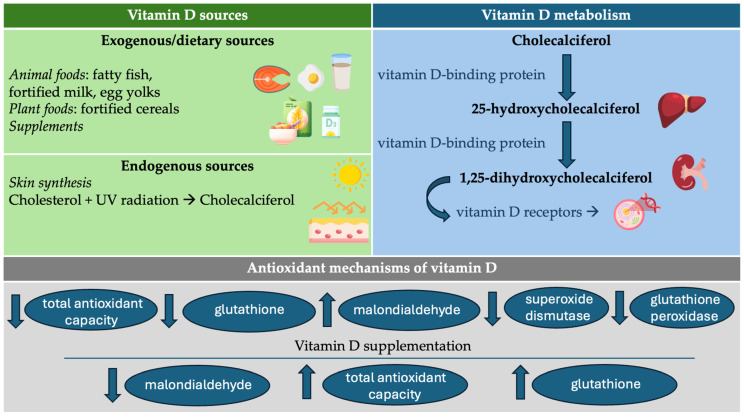
Vitamin D sources, synthesis, metabolism, and antioxidant mechanisms. Abbreviations. UV, ultraviolet.

The recently identified as non-canonical pathways of vitamin D activation are initiated by CYP11A1, an essential enzyme in steroidogenesis present in various organs and tissues, including the skin, peripheral tissues, adrenal glands, placenta, gastrointestinal tract, and diverse cancer cells [35]. CYP11A1, the first enzyme in steroidogenesis or the non-canonical pathway, is responsible for hydroxylating and cleaving the side chain of cholesterol to produce pregnenolone [36]. This enzyme also has the capability to hydroxylate and cleave the side chain of 7DHC and hydroxylate the side chains of vitamin D_3_ and D_2_ without cleavage, involving a complex process with several cytochrome P450 (CYP) enzymes and generating numerous hydroxyderivatives. Additionally, CYP11A1 can activate tachysterol and lumisterol through sequential hydroxylation, resulting in multiple hydroxylated forms [37,38,39]. By binding to the VDR genomic site, these compounds can modulate gene expression like 1,25-dihydroxyvitamin D_3_. Furthermore, hydroxyderivatives of vitamin D_3_, including those derived from tachysterol and lumisterol, interact with other nuclear receptors such as the aryl hydrocarbon receptor (AhR), retinoic acid orphan receptors (ROR)α and γ, and liver X receptors (LXR)α and β, thereby influencing their expression and activity [40]. Vitamin D sources, synthesis, and metabolism are summarized in Figure 1.

### 3.2. Vitamin D Deficiency and Its Implications

Vitamin D deficiency has emerged as a significant global health concern, affecting approximately 1 billion individuals worldwide and presenting substantial health risks [41]. The prevalence of vitamin D deficiency is estimated to range between 60% and 80%, with even higher rates observed among the elderly population [42]. The circulating concentration of 25-hydroxyvitamin D serves as a comprehensive indicator of overall vitamin D status, as it reflects the levels derived from various sources and has a half-life of approximately two months, making it the most reliable measure of vitamin D levels in the body [43]. Several factors contribute to the risk of 25-hydroxyvitamin D deficiency, including race, elevated body mass index, seasonal variations (particularly during winter months with reduced daylight), residence in higher latitudes, and dietary insufficiencies in vitamin D [44]. Notably, obesity is associated with lower circulating levels of vitamin D due to factors such as volumetric dilution and sequestration in adipose tissue [45]. Consequently, vitamin D deficiency is linked to an increased risk of numerous diseases and clinical conditions [46].

The identification of VDRs in virtually all human cells has expanded our understanding of vitamin D’s potential roles in various biological processes [47]. Vitamin D is known to regulate the expression of over 1000 genes in the human genome, accounting for approximately 5% of all protein-coding genes [48]. Its active form acts as a potent inhibitor of cell proliferation while promoting cell differentiation and apoptosis in cancer cells [49]. VDRs are not confined to tissues involved in bone metabolism but are also present in numerous other tissues, including the brain, prostate, breasts, and immune cells. This widespread distribution of receptors associates vitamin D with a range of chronic diseases [50]. Vitamin D deficiency has been implicated in a variety of clinical conditions. For example, it is linked to cardiovascular disease by diminishing pro-inflammatory cytokines production [51], type 2 diabetes through its role in stimulating insulin production and regulating genes involved in glucose metabolism [52], neuropsychiatric disorders, and endocrinopathies [53]. Additionally, it affects tumor development and immune diseases by modulating the number of embryonic hematopoietic stem cells [54], influencing the differentiation of myeloid progenitor cells into monocytes and granulocytes [55], and affecting the differentiation of monocytes into dendritic cells and macrophages [56]. Furthermore, vitamin D has recently been recognized for its antioxidant properties [57]. Similar to 1,25-dihydroxyvitamin D_3_, the principal hormonally active form of vitamin D_3_, hydroxymetabolites derived from CYP11A1 also demonstrate anticancer properties, including antiproliferative and pro-differentiation effects, as well as immunomodulatory functions [58]. Additionally, these CYP11A1-derived vitamin D_3_ hydroxyderivatives have been found to offer protection against skin damage by mitigating UVB-induced DNA damage and reducing levels of ROS [59]. 

## 4. Antioxidant Functions of Vitamin D

In 1993, Helen Wiseman demonstrated that vitamin D is an antioxidant vitamin [60]. Since then, many more studies have been conducted, and vitamin D mechanisms have become increasingly clear [61]. Namely, vitamin D is known to enhance antioxidant gene expression via binding to the vitamin D response element [62]. Specifically, vitamin D deactivates the nuclear transcription factor kB (NF-KB) by increasing the expression of IkB and decreasing the phosphorylation of IkB-α. It suppresses the expression of the nicotinamide adenine dinucleotide phosphate (NADP) enzyme and prevents the accumulation of advanced glycation end-products, which may contribute to the suppression of OS [63]. However, one of the main mechanisms that explain vitamin D effects on antioxidant enzymes is the indirect regulation of the expression of nuclear factor erythroid 2-related factor 2 (Nrf2)-regulating also the expression of other relevant genes to the ROS signaling pathway function, such as klotho [64], which in turn increases the production of antioxidant enzymes [65]. Mainly, the Nrf2 induces the expression of vitagenes, which are cytoprotective genes [66]; these are essential for cellular defense mechanisms such as redox equilibrium and detoxification. Furthermore, these proteins control the expression of a variety of proteins, including heat-shock proteins, which have been shown to enhance cytoprotection in a number of diseases and processes, including cancer, inflammation, neurodegenerative diseases, and aging. [67]. Consequently, vitamin D has been seen to affect different parameters related to antioxidant status, thus impacting on the total antioxidant capacity (TAC) [68]. In this context, vitamin D scavenges physiologically relevant free radicals by increasing SOD levels which react with oxygen radicals [69]. Furthermore, an increase in the intracellular pool of reduced GSH has been also observed, partially through upstream regulation of the GR and glutamate-cysteine ligase (GCL) genes, GCL being a vital enzyme in GSH production [70]. The expression of thioredoxin reductase 1 (TXNRD1) is also induced by vitamin D. As well as reducing thioredoxin, TXNRD1 reduces glucose-6-phosphate dehydrogenase (G6PD), which generates NADPH to regenerate glutathione (GSH) [71]. These functions of vitamin D may be observed in different cells. For instance, vitamin D exerts an antioxidant effect on monocytes by upregulating GR and GCL, thereby reducing oxygen radicals, including ROS [72]. 

Additionally, vitamin D appears to play an essential role in protecting mitochondria and preventing lipid peroxidation through the regulation of inflammatory processes, ROS concentrations, and mitochondrial antioxidant secretion through the cell signaling pathway [70]. ROS generation may be boosted by changes in mitochondrial energy metabolism during vitamin D deficiency, along with decreased antioxidant defenses [73]. Moreover, hyperglycemia disturbs electron transfer and mitochondria activation, causing excessive ROS production via the polyol pathway [51]. Having a suboptimal vitamin D status fails to subdue OS conditions, augmenting intracellular oxidative damage and the apoptosis rate. Indeed, intracellular Nrf2 is inversely correlated with mitochondrial ROS accumulation and OS escalation [71]. In addition to improving OS, cytokine production, and proinflammatory status, vitamin D can normalize mitochondrial dynamics [74]. A further benefit of vitamin D is that it inhibits lipid peroxidation and exerts antioxidant effects through the alteration of antioxidant enzymes in the cell membrane [51]. Malondialdehyde (MDA) levels, which are considered a marker of lipid peroxidation, could be lowered via vitamin D supplementation [75]. As a result of their structural similarity, vitamin D and cholesterol, as well as ergosterol, are known to possess antioxidant properties in the membrane [60]. Consequently, vitamin D could serve as an antioxidant for the cellular membrane as it inhibits the oxidation of lipids induced by iron in brain liposomes [76].

Hydroxymetabolites of vitamin D_3_ derived from CYP11A1, such as 20-hydroxyvitamin D_3_ (20(OH)D_3_) and 20,23-dihydroxyvitamin D_3_ (20,23(OH)_2_D_3_), have been observed to exhibit anti-oxidative properties comparable to those of the classical active form of vitamin D_3_. The anti-oxidative effects of active vitamin D forms can be mediated through the activation of NRF2 and p53 signaling pathways, involving interaction with the VDR and inverse agonism on RORγ, which subsequently attenuates Th17 responses [29]. Consistent with this, CYP11A1-derived vitamin D_3_ hydroxyderivatives have been shown to protect cultured human keratinocytes and melanocytes from UVB-induced damage. This protection is characterized by reduced production of ROS, H_2_O_2_, and nitric oxide (NO), as well as an increased expression of genes encoding enzymes involved in OS defense [77]. Furthermore, hydroxylated forms of lumisterol have been demonstrated to induce the expression of several genes associated with protection against OS [35].

**Figure 2:**
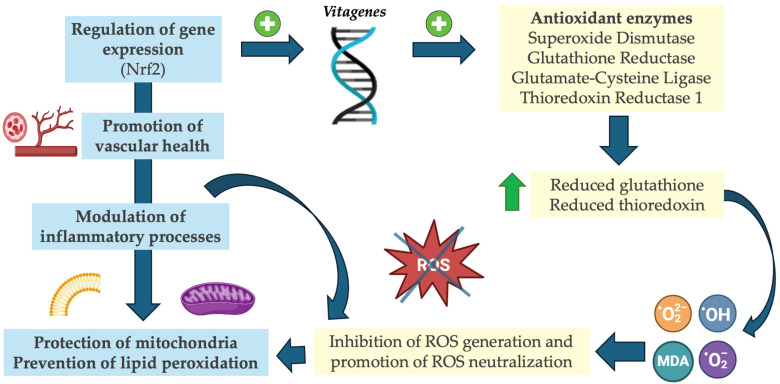
Antioxidant functions of vitamin D. Abbreviations: MDA, Malondialdehyde; Nrf2, nuclear factor erythroid 2-related factor 2; ^•^OH, hydroxyl radicals; ^•^O_2_^−^, superoxide radicals; ^•^O_2_^2−^, hydrogen peroxide; ROS, reactive oxygen species.

Recently, vitamin D has garnered increased attention as a non-enzymatic antioxidant compound. Vitamin D exerts its antioxidant effects through several mechanisms, including the inhibition of free radical generation by nitric oxide synthase (NOS) and gamma-glutamyl transpeptidase (GGT) [78]. Nevertheless, there is variability in the literature concerning the antioxidant effects of vitamin D. A systematic review of clinical trials involving non-pregnant individuals indicated that beneficial effects on OS parameters, such as reductions in MDA levels and increases in GSH, were observed only at high doses of 100,000–200,000 IU per month [79]. While many studies report a reduction in MDA levels with oral vitamin D supplementation, the findings are inconsistent, with some studies failing to demonstrate positive effects [80]. These discrepancies may be attributed to variations in the study design, participant characteristics, vitamin D dosage, administration route, and study duration [81]. The principal antioxidant mechanisms of vitamin D are reviewed in Figure 2.

### 4.1. Evidence from Clinical and Experimental Studies

#### 4.1.1. Cell Culture Studies and Animal Models

It has been documented that vitamin D has a substantial protective role against cellular stress and may function as an antioxidant in different cell types like hepatocytes [82]. Vitamin D has also demonstrated antioxidant properties, notably in protecting cardiomyocytes from toxicity induced by aluminum phosphide [83]. Additionally, it has been shown to downregulate intracellular adhesion molecule-1 (ICAM-1) expression in peripheral blood mononuclear cells following exposure to tumor necrosis factor-alpha (TNF-α). In monocytes, vitamin D is able to reduce intracellular adhesion molecule (ICAM-1) expression after TNF-α exposure [84], and improves antioxidant properties in these cells by decreasing lipid peroxidation and increasing oxidative capacity in monocytes [60,85]. Furthermore, by mitigating OS in endothelial cells, vitamin D may contribute to the inhibition of atherosclerosis [86]. In fact, supplementation with vitamin D has been related to a significant reduction in oxidized lipid markers. 

Studies conducted on animal models have investigated the antioxidant properties of vitamin D. In this line, using a rat model of chronic immobilization-induced cardiac dysfunction, supplementation with vitamin D resulted in a significant dose-dependent reduction in OS, which was achieved through an increase in the activity of antioxidant enzymes improving directly the mitochondrial function [87]. In a mouse model of triple-negative breast cancer, vitamin D supplementation mitigated doxorubicin-induced cardiotoxicity by reducing ROS and mitochondrial damage, while preserving the anticancer efficacy of doxorubicin [88]. Additionally, vitamin D has been shown to offer protection against dextran sulfate sodium-induced colitis by enhancing the activity of 3-hydroxy-3-methylglutaryl-CoA synthase 2, an enzyme implicated in vitamin D-mediated defense against OS and inflammation. These findings suggest that vitamin D may provide a potential therapeutic strategy for alleviating OS and inflammation associated with colitis [89]. 

On the other hand, different studies have pointed out the potential of vitamin D supplementation for improving food production. In dairy cows, where there are critical moments that produce an increase in OS that can trigger a pathology, such as the change from lactation to feed, the concentration of vitamin D correlated with antioxidant potential [90]. Likewise, although the administration of a regular dosage of 2000 IU of vitamin D within the diet of weaned piglets did not achieve optimal antioxidant capacity or immune function [91], vitamin D supplementation in growing-finishing pigs, fed with a low-phosphorus diet, resulted in increased serum levels of SOD and glutathione peroxidase (GSH-Px), alongside decreased serum levels of bone-specific alkaline phosphatase. Vitamin D supplementation also elevated mucosal GSH-Px activity in the duodenum and ileum and TAC and SOD activity in the longissimus dorsi muscle. Furthermore, vitamin D significantly improved mRNA levels of copper/zinc SOD and modified the mRNA levels of Keap1 and Nrf2 [92]. In a study assessing the impact of vitamin D on growth performance, antioxidant enzyme activity, and antimicrobial capability in juvenile grass carp, six supplemental levels of vitamin D (0, 300, 600, 1200, 2400, and 4800 IU/kg diet) were tested. Supplementation with vitamin D at levels ranging from 300 to 2400 IU/kg significantly improved growth performance and antioxidant enzyme activity in fish livers compared to the vitamin D deficiency group [93].

#### 4.1.2. Human Studies

While the impact of vitamin D on mitigating OS in cellular and animal models has been consistently demonstrated, clinical outcomes in human subjects have yielded mixed results. This variability may be attributed to factors such as differences in dosage, treatment duration, and clinical settings [94]. Vitamin D has been investigated for its potential therapeutic effects across a range of diseases in humans. There have been several studies exploring the antioxidant properties of vitamin D within the context of COVID-19 [95,96,97]; however, that is not the main focus of this review. 

In order to illustrate the importance of vitamin D in OS, a wide range of pathologies are shown below. 

A recent meta-analysis suggests that antioxidants could represent an effective and safe treatment for patients with atopic dermatitis, particularly when combined with oral vitamin D and topical vitamin B_12_ [98]. In a randomized, double-blind, placebo-controlled trial involving 75 children aged 6–12 with attention deficit hyperactivity disorder, children were allocated to receive either vitamin D (2000 IU) or a placebo for three months. The supplementation with vitamin D did not yield significant benefits on biomarkers of OS [99]. 

In patients with Behçet’s disease, an association has been observed between low levels of vitamin D and increased OS, as evidenced by elevated MDA and NO levels, alongside decreased levels of GSH, SOD, and catalase activities, as well as TAC. Therefore, fortified foods, beverages, or vitamin D supplements may potentially ameliorate the severity of Behçet’s disease and reduce OS [70].

A recent umbrella meta-analysis indicates that vitamin D supplementation in adults may lead to reductions in C-reactive protein (CRP), MDA, and TNF-α levels across various health conditions. Consequently, vitamin D could be studied as a supplementary therapy for alleviating inflammation and OS [62]. 

Furthermore, the impact of supplementation with vitamin D on inflammatory biomarkers and OS in pregnant women has been assessed in another systematic review, which reported increased levels of vitamin D, TAC, and GSH, along with decreased MDA concentrations following supplementation [100].

### 4.2. Antioxidant Properties of Vitamin D in Chronic Diseases 

#### 4.2.1. Cardiovascular Health and Endothelial Function

VDRs have been identified in the heart, suggesting potential intracrine or paracrine roles in the cardiovascular system [87]. In fact, Vitamin D use has been recommended as an adjunct therapy in the treatment of inflammatory diseases and cardiometabolic conditions [21]. It exerts a vasoprotective effect by reducing OS-induced endothelial dysfunction and regulating the bioavailability of NO [101]. OS is a key contributor to endothelial dysfunction, damaging the endothelial lining and impairing its ability to control the blood flow and preserve vascular health. The antioxidant effects of vitamin D protect endothelial cells from oxidative damage, preserving their functionality and promoting vascular health [102]. ROS are well-known contributors to cardiovascular disease, being the excessive release of these free radicals from various intracellular sources related to disturbances in the normal function of endothelial cells and cardiac myocytes. Hence, OS is implicated in the development of different diseases, such as cardiac hypertrophy and heart fibrosis resulting from long-term hypertension [103].

Vitamin D regulates the endothelial cell function by interacting with the endothelial NOS pathway. The NOS is an enzyme responsible for producing NO in the endothelium. Vitamin D enhances NOS expression and activity, thereby promoting NO production and supporting the optimal endothelial function. The NOS pathway is essential for maintaining vascular homeostasis and preventing endothelial dysfunction [104].

#### 4.2.2. Diabetes and Insulin Resistance

Besides hyperinsulinemia and hyperglycemia, diabetes is characterized by increased free radical formation and decreased TAC [105]. Vitamin D supplementation protocols that normalize blood vitamin D concentrations may improve glycemic control parameters and reduce OS in diabetic patients [106]. In this regard, vitamin D plays a significant role in managing OS by modulating the Nrf2 gene expression and influencing the pathogenesis of diabetes through its effects on the β-cell function, peripheral insulin sensitivity, or both [90]. Furthermore, vitamin D has been effective in improving the inflammatory response and OS, as well as maintaining glucose metabolic homeostasis in patients with gestational diabetes mellitus [107]. Moreover, a decrease in serum MDA levels and an increase in TAC following vitamin D supplementation have also been reported in patients with diabetes and vitamin D deficiency [108]. A high dosage of vitamin D supplementation significantly elevated TAC and GSH levels in women with gestational diabetes [109]. Conversely, vitamin D supplementation did not affect the majority of antioxidant enzyme activities in diabetic mice. By increasing intracellular zinc concentrations, oxidative damage was indirectly mitigated [110]. In older people with diabetes, blood vitamin D concentrations were inversely correlated with various circulating OS biomarkers, including advanced oxidation protein products, low-density lipoprotein oxidation susceptibility, and NO metabolites, which were indirectly mitigated by increasing intracellular zinc concentrations [111]. Furthermore, antioxidant supplementation may not be beneficial to all individuals with diabetes, as their response to vitamin D supplementation may vary depending on their VDR FokI genotype [112].

#### 4.2.3. Neurodegenerative Diseases

Vitamin D has a significant role in neuronal development, encompassing processes such as proliferation, differentiation, migration, and axonal growth. It stimulates GGT activity, thereby preventing hippocampal damage [113]. Decreased TAC and increased ROS lead to damage in DNA, proteins, and membrane fatty acids, ultimately resulting in cell neurodegeneration, apoptosis, and volumetric changes in the brain [114]. The brain is particularly vulnerable to OS and damage due to its high oxygen consumption, deficiency in antioxidant enzymes, and high content of polyunsaturated fatty acids, which are prone to oxidation. Vitamin D regulates the oxidant–antioxidant balance by increasing intracellular antioxidant concentrations and reducing OS by scavenging excess free radicals [115]. As an antioxidant, vitamin D controls brain detoxification processes through the upregulation of GGT activity [78]. The neuroprotective role of vitamin D is extremely important due to its role in regulating calcium homeostasis in brain cells [116]. By treating hippocampal cells with high doses of vitamin D, they were protected from age-related excitotoxicity, resulting in a significant downregulation of L-type voltage-sensitive calcium channels [117,118], which are normally expected to surge in aging hippocampal cells and damaged cells [119,120]. A total of 74 genes and 36 proteins are regulated by vitamin D, and it also participates in the post-transcriptional control of voltage-sensitive calcium channels of the L-type [121,122].

Furthermore, after traumatic brain injury, where there is an increase in autophagy and an inhibition of Nrf2 signaling leading to uncontrolled OS, vitamin D treatment significantly reduces neurological deficits and histopathological changes [123]. 

This study demonstrated that vitamin D effectively protected the mitochondria in a Parkinson’s disease model from dysfunction and oxidative damage, suggesting that it could be used as a therapeutic agent in the treatment of neurodegenerative disease [124]. Vitamin D supplementation reduced MDA levels, suggesting a protective effect that is at least partially associated with the prevention of oxidative damage production [125]. Vitamin D could play also an important role in modulation inflammation in the hypertensive brain, where the renin-angiotensin system (RAS) exerts pro-inflammatory and pro-oxidant effects. Vitamin D modulates the RAS, and it could be responsible for a neuroprotective effect described in this pathology [126]. Indeed, vitamin D deficiency has been reported to downregulate antioxidant enzymes, including SOD and GSH-Px. Vitamin D also inhibits NOS, thereby reducing NO production, while increasing GSH levels in the brain [127]. Regarding Alzheimer’s disease, vitamin D may ameliorate cognitive deficits induced by aluminum-chloride-D-galactose through the upregulation of the calcium/calmodulin-dependent protein kinase-kinase 2-AMPK/Sirtuin1 pathway. This mechanism restores the normal mitochondrial function and reduces inflammation and OS [128]. Furthermore, given that OS can initiate microglial activation, which contributes to the pathophysiology of Alzheimer’s disease, vitamin D’s antioxidant properties through the activation of Nrf2 may be associated with its neuroprotective properties against the development and progression of Alzheimer’s disease [129]. Moreover, by acting as an antioxidant, it has been shown to reduce OS biomarkers in rats with Alzheimer’s disease hippocampus and serum [130], while vitamin D has been shown to protect the cortex from OS by increasing the levels of SOD, GSH, and TAC, as well as reducing MDA levels in mice [131].

#### 4.2.4. Chronic Kidney Disease

Dietary antioxidants, such as vitamin D, which scavenge free radicals, are garnering significant interest as potential therapeutic agents for patients with chronic kidney disease (CKD) [132]. CKD is a global health issue affecting 5–10% of the population worldwide, and is characterized by an elevated inflammatory state, impaired immune response, and OS [133]. In individuals with CKD, vitamin D has been demonstrated to mitigate OS by upregulating Nrf2 and activating the antioxidant response element, highlighting a non-traditional regulatory function of the vitamin D pathway in the context of CKD [94]. Specifically, the expression of Nrf2 and serum levels of antioxidant enzymes have been observed to increase in individuals with end-stage renal disease [134]. Furthermore, vitamin D enhances the expression of Klotho proteins, a family involved in antioxidant production that is predominantly expressed in the kidneys. Klotho proteins have been shown to reduce OS by modulating the NF-kB expression and preventing other deleterious effects of CKD [135]. Conversely, vitamin D deficiency was not found to play a significant role in the increased OS state observed in pre-dialysis CKD patients, indicating the multifactorial nature of this condition [136].

#### 4.2.5. Cancer

Age-related reductions in vitamin D synthesis and VDR expression are linked to age-related diseases such as cancer [137]. Increased vitamin D intake and higher blood vitamin D concentrations have been proposed as interventions to mitigate conditions such as cancer [3]. Despite this, human studies on the effects of vitamin D treatment on circulating antioxidant and OS biomarkers, as well as cancer incidence and prognosis, have produced conflicting results [138]. Vitamin D contributes to tumorigenesis through various pathways, including the upregulation of antioxidant enzymes and inhibition of lipid peroxidation [138]. VDR activation may inhibit p53 signaling pathway activation, thereby promoting the proliferation, invasion, and metastasis of esophageal squamous cell carcinoma through OS mechanisms [139]. Moreover, osteosarcoma, which is characterized by high ROS production, relies significantly on OS for its pathogenesis. Vitamin D has been found to inhibit osteosarcoma, suggesting therapeutic applications not only for osteosarcoma patients but also for the broader aging population by mitigating OS [140]. Another possible mechanism involves vitamin D deficiency impairing mitochondrial respiration by downregulating complex I expression in the electron transport chain. This downregulation reduces the formation of adenosine triphosphate, thereby increasing cancer risk. Furthermore, compromised electron transport chain activity leads to elevated ROS production and heightened OS [141]. Finally, CYP11A1-derived secosteroids exhibit anti-cancer activities that vary depending on the specific cell type and lineage involved [142]. In this context, vitamin D hydroxymetabolites and lumisterol, along with their respective receptors, play a pivotal role in the prevention of skin cancer by activating protective anti-cancer pathways, such as the Nrf2-antioxidant response and p53 phosphorylation. Furthermore, these compounds promote DNA repair mechanisms that safeguard human keratinocytes from DNA damage [143]. UVR enhances the production of ROS, which may have harmful effects on the skin, and induces the tumor suppressor protein p53 as part of the cellular response to DNA damage. This process increases the risk of melanoma, a risk that may be mitigated by the antimelanoma activity of CYP11A1-derived secosteroids [27].

## 5. Future Directions

Despite the progress made in the study of molecular mechanisms related to Vitamin D’s antioxidant activity, they are not completely understood at present. In this regard, investigating the precise molecular pathways through which vitamin D exerts its antioxidant effects is needed, being one of the promising markers the gene expression of the VDR and its effect on different processes [144]. Additionally, another interesting field would be to explore vitamin D’s impact on the mitochondrial function and its role in reducing mitochondrial OS. This includes studying how vitamin D affects the mitochondrial biogenesis, dynamics, and function [145].

On the other hand, it is necessary to conduct large-scale and long-term clinical trials to evaluate the efficacy of vitamin D supplementation in preventing and managing OS-related diseases, as well as to identify and validate specific biomarkers of OS that can be reliably used to measure the antioxidant effects in human subjects [146]. Furthermore, it would be necessary to determine the optimal dosage and form of vitamin D supplementation to achieve significant antioxidant effects, without adverse outcomes and taking into account factors like age, sex, ethnicity, genetic variations, and pre-existing health conditions, leading to more tailored supplementation recommendations [50]. Similarly, the development and efficacy of vitamin D analogues and derivatives that might have enhanced antioxidant properties or reduced side effects compared to natural vitamin D should be investigated [147].

In the context of CYP11A1-derived vitamin D and lumisterol metabolites, exploring the therapeutic potential of vitamin D hydroxyderivatives in the treatment of chronic diseases associated with OS presents promising new avenues for medical intervention. However, the precise molecular mechanisms through which these metabolites modulate OS remain only partially understood. Future research should prioritize the identification of specific signaling pathways and target genes that are regulated by these metabolites. This includes examining their interactions with non-classical receptors and elucidating how these interactions affect cellular redox homeostasis [37,148,149].

**Figure 3:**
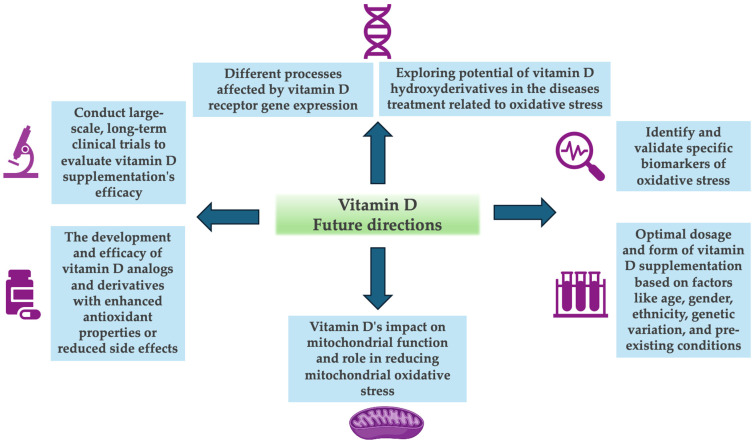
Main future directions for vitamin D.

Finally, the relationship between vitamin D status and the progression of chronic diseases characterized by high OS levels, providing insights into potential therapeutic roles for vitamin D like cardiovascular diseases, type 2 diabetes, and CKD should be studied more in greater depth [150]. Likewise, vitamin D’s role in modulating the immune system’s response to OS, particularly in conditions where inflammation and OS are closely linked, such as autoimmune diseases, should be investigated [151]. Moreover, conducting studies to assess the impact of vitamin D on cognitive function and the prevention of cognitive decline through its antioxidant properties could clarify its neuroprotective effects [152]. An overview of vitamin D’s future directions in antioxidant activity is shown in Figure 3.

## 6. Conclusions

Vitamin D has been seen to affect different parameters related to antioxidant status. This function is mainly associated with increased levels of antioxidant enzymes and the regulation of the expression of Nrf2 and Klotho. These antioxidant properties present a compelling case for vitamin D’s role in promoting cellular health and preventing oxidative damage. Future research should focus on elucidating the precise molecular pathways involved and exploring the therapeutic potential of CYP11A1-derived vitamin D and tachysterol and lumisterol metabolites in the skin and vitamin D supplementation in conditions characterized by elevated OS as chronic diseases. This expanded understanding could pave the way for novel interventions aimed at leveraging their antioxidant capabilities to enhance health outcomes and potentially reduce chronic diseases’ impact on human health.

## Data Availability

Not applicable.
